# Feature Selection and Validation of a Machine Learning-Based Lower Limb Risk Assessment Tool: A Feasibility Study

**DOI:** 10.3390/s21196459

**Published:** 2021-09-27

**Authors:** Swagata Das, Wataru Sakoda, Priyanka Ramasamy, Ramin Tadayon, Antonio Vega Ramirez, Yuichi Kurita

**Affiliations:** 1 Graduate School of Advanced Science and Engineering, Hiroshima University, 1-4-1, Kagamiyama, Higashi-Hiroshima City, Hiroshima 739-8527, Japan; watarusakoda@hiroshima-u.ac.jp (W.S.); priyankaramasamy@hiroshima-u.ac.jp (P.R.); a.vegaramirez76@gmail.com (A.V.R.); ykurita@hiroshima-u.ac.jp (Y.K.); 2 School of Computing, Informatics & Decision Systems Engineering, Arizona State University, Tempe, AZ 85281, USA; ramint@axosoft.com

**Keywords:** artificial neural network (ANN), Random Forest regressor, skill assessment, squat, one-leg standing, locomotive syndrome

## Abstract

Early and self-identification of locomotive degradation facilitates us with awareness and motivation to prevent further deterioration. We propose the usage of nine squat and four one-leg standing exercise features as input parameters to Machine Learning (ML) classifiers in order to perform lower limb skill assessment. The significance of this approach is that it does not demand manpower and infrastructure, unlike traditional methods. We base the output layer of the classifiers on the Short Test Battery Locomotive Syndrome (STBLS) test used to detect Locomotive Syndrome (LS) approved by the Japanese Orthopedic Association (JOA). We obtained three assessment scores by using this test, namely sit-stand, 2-stride, and Geriatric Locomotive Function Scale (GLFS-25). We tested two ML methods, namely an Artificial Neural Network (ANN) comprised of two hidden layers with six nodes per layer configured with Rectified-Linear-Unit (ReLU) activation function and a Random Forest (RF) regressor with number of estimators varied from 5 to 100. We could predict the stand-up and 2-stride scores of the STBLS test with correlation of 0.59 and 0.76 between the real and predicted data, respectively, by using the ANN. The best accuracies (R-squared values) obtained through the RF regressor were 0.86, 0.79, and 0.73 for stand-up, 2-stride, and GLFS-25 scores, respectively.

## 1. Introduction

The locomotor system of the human body performs complex processes of control and coordination. Our locomotor system’s efficiency is affected by various factors such as accidental injury, aging, arthritis, osteoporosis, and most importantly a sedentary lifestyle. During the COVID-19 pandemic, we have observed an increase in sedentarism or physical inactivity, thus threatening both physical and mental health [1]. Hospitalization is also considered as a contributing factor in causing reduced mobility, resulting in functional decline in older adults during discharge [2]. Reduced physical activity and sedentary behavior predominantly affect older adults due to increased fall risk [3]. The consequences of unexpected falls and related costs constitute substantial concern in the modern world [4]. Moreover, most Asian and European countries are experiencing a significant increase in the proportion of their geriatric population [5]. This population trend is more common in countries such as Japan, where the elderly constitutes over one-third of the national population. Physical and health examination can be considered as global requirements to reduce accidents caused by musculoskeletal degeneration and other locomotive health effects of aging and sedentarism. The lower extremity facilitates the entire body’s mobility with the ankle, knee, and hip joints depending on stability and control. Therefore, a timely risk assessment of the lower-limb may significantly reduce the risk of falls and increase awareness, making assessment of the stability of human locomotion a vital research area owing to reducing the risk of falls and unstable gait.

In 2007, the Japanese Orthopaedic Association (JOA) introduced the concept of *Locomotive Syndrome (LS)*. They defined it as a condition of mobility disorder that results from the degeneration of locomotive organs such that nursing care becomes a requirement. With a high proportion of older adults, most of the Japanese population experienced LS during the latter part of their lives. According to a survey conducted by the Japanese Ministry of Health, Labor, and Welfare in 2016, 6.32 million people requested nursing care for the elderly, which is more than twice compared to the count 15 years ago [6]. The risk level of LS can be evaluated by a self-reported assessment test proposed by the JOA, which is widely known as the Short Test Battery Locomotive Syndrome (STBLS) [7]. This test makes it possible to identify LS, which is otherwise challenging in terms of diagnosis. Identifying early stage LS enables us to slacken expenses and motivate diagnosed patients to remain active. Even though the STBLS test is a widely used tool for diagnosing LS, it requires the presence of a therapist or supporter to perform the evaluation. In addition, human-based measurements are subject to inadvertent errors and require measurement tools. Therefore, self-sustained yet accurate measurement techniques show better chances of acceptance by the present society.

In this paper, we propose the utilization of skeletal data obtained by using the Intel RealSense depth camera to predict the occurrence of Locomotive Syndrome (LS) by using Machine Learning (ML) classification techniques. The usage of Intel RealSense is an interesting novelty in this work in terms of motion capture showing a distinctive approach compared to other standard Kinect-based analysis utilized in prior literature. The Intel RealSense depth camera provides a frame-rate of 90 Frames Per Second (FPS) and depth resolution of 1280 × 720 thus, outperforming the Kinect V2 (FPS: 30, depth resolution: 512 × 424). The RealSense camera is compact and low-priced and yet, its remaining specifications are comparable to Kinect V2. We used the Intel RealSense camera to collect skeletal movement during two exercise tasks, namely squat and one-leg standing. One of the major contributions of this paper is the calculation of 13 input parameters from the raw skeletal data obtained during both exercise tasks that are later used to train the classification algorithms. The predefined output data for training the ML classifiers are acquired by using the stand-up, 2-stride, and GLFS-25 scores of the STBLS test. Two ML classifiers facilitated high accuracy while verifying the feasibility of the proposed set of input and output ML parameters, namely Artificial Neural Network (ANN) and Random Forest (RF) regressor.

## 2. Related Work

Several clinically approved mobility assessment methods have been proposed in order to evaluate gait, balance, and transfer abilities in older adults. In terms of assessment types, these tests can be categorized into performance-based, performance and judgment-based, and self-reported. The top two performance-based assessment methods are Timed Up and Go (TUG) [8] and Soft Physical Performance Battery (SPPB) [9]. The top two performance and judgment-based measures are Tinetti Performance Oriented Mobility Assessment (Tinetti-POMA or TMT) [10] and Berg Balance Scale (BBS) [11]. In terms of the specific aim of evaluation, there are tests aimed at assessing balance and walking [8], overall mobility [12], gait speed [13], balance and transfers [11], postural stability [14], functional stability [15], and fall risks [16]. Clinical methods of risk assessment have limitations such as requiring a therapist’s support, low precision due to potential human errors during measurement, and fatigue in both therapists and patients. Patients cannot carry out clinical evaluations other than self-reported risk assessment methods at home without a therapist’s involvement. Additionally, self-reported tests have a high possibility of being affected by human bias and error.

Sensor-based evaluation for assessing limb mobility has also gained popularity during the past years. These techniques can be broadly categorized into on-body tracking and environment tracking [17]. On-body tracking involves the use of sensors and trackers attached to different body parts aimed at tracking movements. Environment tracking is performed through monitoring systems such as cameras placed at a distance from the user to track their movements. First, we consider some examples of on-body tracking evaluation methods. Accelerometers are frequently used as on-body trackers for gait analysis and detection [18]. Wii Balance board is another commonly used device. Yamada et al. used it for fall risk assessment in healthy community dweller older adults [19]. A novel infrared laser device for measuring multilateral stepping-performance parameters was introduced by Nishiguchi et al. for identifying fall-risk in elderlies [20]. Takeda et al. predicted footprints from the sole pressure distribution during walking and developed a diagnostic system using features such as the mean absolute error [21]. Next, we mention some environment tracking type evaluation techniques. Cary et al. presented a Kinect-based system combined with ANN classifiers for physiotherapy assessment. The primary aim of this research was to provide quantitative data of the patients to the physiotherapists to ensure the ability to monitor and record both quantitative and qualitative skeletal data during physiotherapy sessions [22]. Another Microsoft Kinect-based fall-risk assessment method while performing tasks drawn from clinical balance scales is proposed in [23]. A supervised classifier is deployed for classification in this study. Nintendo introduced Ring Fit Adventure enabled with a motion-sensing resistance ring and a controller strapped to the leg to integrate gaming experiences with physical workouts such as squatting. Such controllers are designed to be wearable and capable of detecting movements wirelessly [24]. However, skill assessment is not the primary rationale behind such systems.

Machine Learning (ML) and Neural Networks (NNs) were introduced in the field of rehabilitation almost three decades ago. Recognizing *healthy* and *pathological* gaits was implemented through an NN-based algorithm in [25]. This study also summarizes different data processing and classification techniques utilized while designing an NN-based clinical tool. The Genetic Algorithm Neural Network (GANN) approach was used to recognize gait patterns and compared with a traditional ANN. The GANN-based approach could classify the data with an accuracy rate of up to 98.7%, while the ANN’s accuracy was limited to 89.7% [26]. Identification of locomotion type and intensity was implemented by measuring the contact data between foot and ground. The data were acquired through a Smart Insole Measurement System (SIMS) and identified by using an ANN. This study mainly detected the type and speed of activities such as running, walking, and climbing up and down [27]. Post-stroke gait classification was performed by using ANN with classification rates of 100% and 86% using knee joint and frontal motion of the hip joint as input parameters, respectively [28]. Principal Component Analysis (PCA), multi-layered FF ANN, and Self Organized Maps (SOM) were used for classifying and clustering gait patterns in normal subjects and patients with lower limb fractures. The study used Vertical Ground Reaction Force (vGRF) as the measured data and showed the highest classification accuracy of 96% [29]. A lower limb joint moment prediction approach using ANN is discussed in [30]. Right lower limb EMG and five joint angle data were used as candidate input variable sets for the ANN during this approach. A single Inertial Measurement Unit (IMU) was used to predict lower limb kinetics and kinematics during walking with the help of ML. This research proposes the prospect of reducing the tradeoff between wearable convenience and data quantity by using ML algorithms utilizing the dynamic characteristics of human movements [31]. A detailed review on the various research studies focused on the automatic recognition of gait patterns in human motor disorders using ML published during the past decade is presented in [32].

Environment tracking systems applied to patient evaluation are comparatively lower in count than on-body tracking. Environment tracking methods are advantageous due to the absence of direct physical contact with patients during the evaluation. Furthermore, on-body tracking requires considerable effort and time for the attachment and calibration of the sensors. Most of the lower limb assessment research approaches include on-body sensors and consider assessing data acquired from subjects who are already advanced pathological patients. Environment tracking-based assessment methods that can identify the early to advanced stages of LS are not yet introduced. Therefore, in this research, we aim to address this issue through a non-contact risk assessment tool. This assessment tool is implemented by using data collected through Intel RealSense depth sensor camera such that the user may be evaluated without physical contact and need for physical therapists.

## 3. Proposed Lower Limb Assessment

It was pointed out in previous research that LS exhibits declining mobility characterized by deteriorated motor functions and mobility deficits, making nursing care mandatory [7]. In addition to aging, factors that may accelerate LS’s progression include lack of exercise, inadequate nutrition, and a sedentary lifestyle. Therefore, LS’s identification at an early stage can create awareness and reduce many healthcare expenses due to its advanced progression. The risk assessment test called STBLS proposed by JOA is a widely used tool for diagnosing LS in Japan. However, a supporter’s presence is still required to identify the performance measures during the evaluation tasks. Moreover, materials such as measuring scales and stools with variable height are required to perform the test. Human bias may also affect the judgment of the assessment results. We need a quantitative self-measurable tool to identify LS’s progression, which can be used by both early and advanced stage LS patients. In this paper, we develop and validate such a tool by using FF ANN and RF regressor. We use the STBLS test as the foundation to acquire outputs used to train our classifiers. To reduce the efforts required to set-up the system, we use an environment tracking system (Intel RealSense D435) to track the skeletal data used as inputs of the classifiers. Squat and one-leg standing are used as the tasks to determine the input features. The frame rate of data acquisition was 30 fps.

### 3.1. Input Features

Feature extraction is a common methodology for creating input datasets of ML classifiers. Raw skeletal data are sorted, and only the relevant features are selected as the inputs according to the classification goal [33]. In the current input dataset, we consider all skeletal data from the lower limb portion to derive the input parameters. Since our main area of importance is the lower limb, we took all data points from this section of the skeletal data. From the previous literature, we derived the understanding that selected data points of the lower limb used to calculate the relevant parameters are suitable to be used as inputs to the classifiers rather than using the entire skeletal data [34].

#### 3.1.1. Squat Features

Squat performance can be classified by using non-invasive skeletal joint data as suggested in previous research. According to Escamilla’s report on knee biomechanics during squatting, knee and hip angular displacement (from standing to squatting) and lateral shakiness are prime indicators [35]. In addition, Center Of Mass (COM) has been widely used to assess balance during exercises involving the lower limbs [36]. Based on such observations, we selected parameters to assess squat performance.

**Figure 1:**
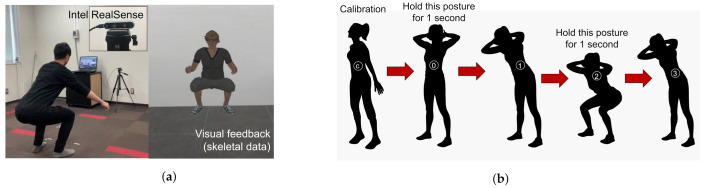
Experiment protocols followed during squat. The posture of arms was not restricted in any experiment. Users were free to keep their arms folded or otherwise as per their preference. (**a**) Squat posture maintained along with the visual feedback given to the user. (**b**) Different states followed during one squat.

Figure 1a represents the outline of the squat measurement system, and Figure 1b shows the states involved in the detection of the squat by the measurement system. The subject starts with a standing posture 2 m away from the depth camera. The depth camera starts measuring the skeletal data. The squat states and posture are shown on the monitor for the user to follow. The algorithm for detecting the squat states is based on previous research by Ramin et al. [34]. The measured squat parameters are shown in Table 1. These parameters are used to calculate the input features of the classifiers.

The knee angles are calculated by using the knee, hip, and ankle joints of the skeleton such that the knee joint is the common point. The hip angles are calculated using the hip, spine, and knee joints of the skeleton such that the hip joint is the common point. The flexion and extension angles are calculated during the states 2 and 0 of squat, respectively, as shown in Figure 1b. The shakiness parameters are calculated using the time delta of lateral position of both knee joints during squat state 2.

Table 2 shows the list of input features derived from the squat parameters. $X_{1},\;X_{2},\;X_{3}$, and $X_{4}$ are obtained by measuring the joint angles during complete upright and squat positions. Values are obtained within the range of [0, 180] and are normalized to the range [0.00, 1.00].

(1) $$X_{1}=abs\left(\theta_{lkf}-\theta_{lke}\right)/105$$

(2) $$X_{2}=abs\left(\theta_{rkf}-\theta_{rke}\right)/105$$

(3) $$X_{3}=abs\left(\theta_{lhf}-\theta_{lhe}\right)/45$$

(4) $$X_{4}=abs\left(\theta_{rhf}-\theta_{rhe}\right)/45$$

The input features $X_{5}$ and $X_{6}$ represent the lateral displacement of the left and right knees during state 2. The maximum raw value (3.50) is used to normalize the data to the range of [0.00, 1.00].

(5) $$X_{5}=1-\frac{s_{lk}}{1000}$$

(6) $$X_{6}=1-\frac{s_{rk}}{1000}$$

The input feature $X_{7}$ represents the Centre Of Mass (COM) smoothness. This value is obtained by using the input features $X_{1}$ to $X_{6}$. For a stable squat, the normalized value is 1.0.

(7) $$\begin{array}{c} X_{7}=\left[1-\frac{0.25*abs(X_{1}-X_{2})}{45}-\frac{0.25*abs(X_{3}-X_{4})}{45}-0.5*\left(abs\left(X_{5}\right)+abs\left(X_{6}\right)\right)-0.7\right]/0.3 \end{array}$$

The input feature $X_{8}$ represents the average time required to complete one full squat. The input feature $X_{9}$ represents the number of squats performed compared to the maximum number of squats performed by any participant.

(8) $$X_{8}=1-\frac{t_{e}-1.5}{0.5}$$

(9) $$X_{9}=\frac{n_{s}}{max(n_{s})}$$

#### 3.1.2. One-Leg Standing Features

One-leg standing is a widely used exercise to train the lower limbs for balance and posture control. It can also be assessed through non-invasive skeletal detection by measuring the standing time and movement trajectory of the waist [37]. Therefore, we used these parameters for evaluating the one-leg standing performance.

**Figure 2:**
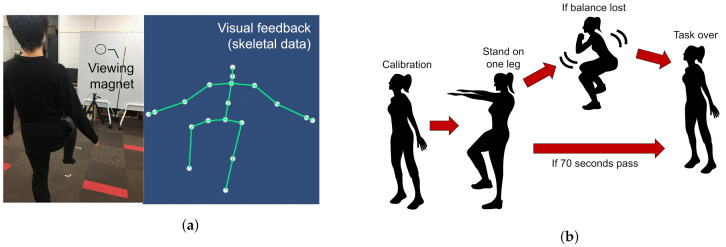
Experiment protocols followed during one-leg standing measurement. (**a**) One leg stance posture maintained along with the visual feedback given to the user. (**b**) Different stages of one-leg standing measurement.

The user starts with an upright standing posture 2 m away from the depth camera. An avatar is displayed on the monitor to replicate the skeletal data of the user’s joints. Figure 2 shows an overview of the one-leg standing measurement system. When the absolute value of the difference between the y-coordinates of both ankles and both knees exceeds a predefined threshold, the system recognizes one-leg standing initiation. If the user loses balance before the stipulated time (70 s), data recording is stopped. The standing time and waist coordinates of the recorded data, as shown in Table 3 are used to calculate the input features $X_{10},\;X_{11},\;X_{12}$, and $X_{13}$ shown in Table 4. For each user, the dominant leg is determined by asking which leg they would prefer to use in kickicking a football. $X_{10}$ and $X_{11}$ correspond to the standing time with the dominant and non-dominant legs, respectively. These values are utilized as additional input features to the neural network. As the stipulated standing time is set to 70 s, $X_{10}$ and $X_{11}$ are obtained in the range of [0, 70]. The raw values are then normalized to [0.00, 1.00].

(10) $$X_{10}=\frac{t_{dl}}{70}$$

(11) $$X_{11}=\frac{t_{ndl}}{70}$$

The input features $X_{12}$ and $X_{13}$ correspond to the waist coordinates’ total trajectory lengths during one-leg standing with dominant and non-dominant legs, respectively. During the measurement, the trajectory length is calculated from the waist coordinate data to obtain the input value. Since there is no fixed maximum value, if the maximum total trajectory length exceeds 1.00, it is normalized to the range of [0.00, 1.00].

(12) $$X_{12}=\frac{\sum_{i=1}^{j}\sqrt{{x_{dl_{i}}}^{2}+{y_{dl_{i}}}^{2}+{z_{dl_{i}}}^{2}}}{j}$$

(13) $$X_{13}=\frac{\sum_{i=1}^{j}\sqrt{{x_{ndl_{i}}}^{2}+{y_{ndl_{i}}}^{2}+{z_{ndl_{i}}}^{2}}}{j}$$

### 3.2. Output Scores

**Figure 3:**
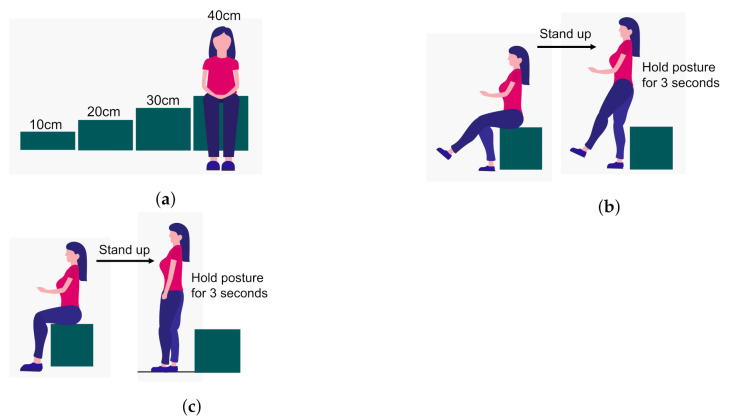
Details of the stand-up test in the form of illustrations [7]. This test is helpful for assessing the subject’s leg strength. (**a**) Different seat heights (10 to 40 cm) used for the stand-up test. (**b**) Stand-up test with both legs on the ground. (**c**) Stand-up test with single leg on the ground.

**Figure 4:**
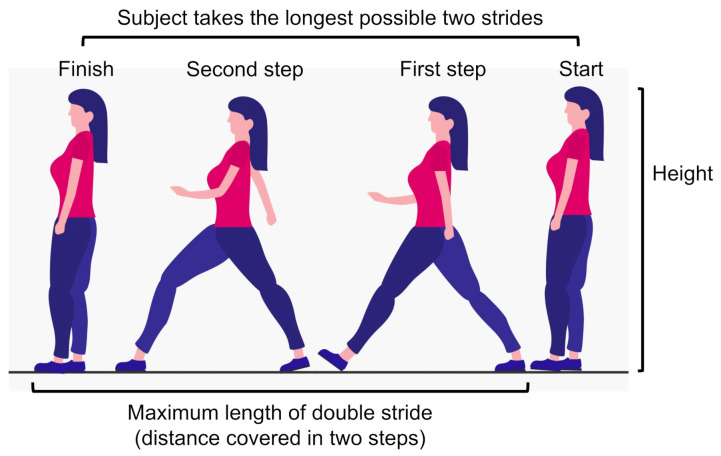
Details of the two-step test in the form of illustrations [7]. This test is used to measure the stride length, which is divided by the subject’s height in order to obtain the final score. This test helps to assess walking ability, muscular strength, balance, gait speed, and lower limb flexibility.

This section illustrates how the three scoring methods of the STBLS test called stand-up, 2-stride, and Geriatric Locomotive Function Scale (GLFS-25) were used to define our classifier’s output scores for quantifying the lower limb risk assessment level. Figure 3 and Figure 4 show the stand-up and 2-stride test details in the form of illustrations.

For stand-up test, the individual is required to stand up from being initially seated on variable height (10, 20, 30, and 40 cm) seats. The action of standing up is observed for conditions of both two-leg and one-leg support. The test is sequenced from easy to difficult levels in which taller seats are less challenging. If the individual can hold the position after standing up for more than 3 s, then it is declared successful. For each seat height, the two-legged test is performed first, and if the test is passed, it is followed by a single-legged test. Scores are assigned on a scale of 0 to 8. A score of zero means the subject is unable to stand and remain balanced in any of the conditions. A score between 1 and 4 means the ability to stand up from heights of 40, 30, 20, and 10 cm using both legs. A score between 5 and 8 means the ability to stand up from heights of 40, 30, 20, and 10 cm using one leg.

The second parameter is based on a test called the two-step test. This test assesses the subject’s gait stability, balance, and lower extremity musculoskeletal strength. In this test, the subject starts by standing on both legs and then takes two steps forward such that the initially grounded foot is used to finish the leap. The subject has to cover as much distance as possible without losing stability or falling. The total length of the stride is measured from the starting point to the endpoint in centimetres, as shown in Figure 4. This distance is then normalized by dividing the value by the subject’s height in centimetres. The resultant ratio represents the value of the second parameter.

The third output score is calculated by a subjective questionnaire called GLFS-25. This questionnaire comprises 25 questions that evaluate the subject’s mobility and motor ability and its effects on social participation. Responses range from 0 to 4, where zero indicates no pain and four indicates high pain and discomfort levels. A lower total score indicates a healthier subject.

The JOA has determined risk calculation based on the individual STBLS parameters and established its relation to the subject’s mobility [38]. We can categorize the lower extremity locomotive risk into three stages zero, one, and two, where zero is healthy, and two is at the highest risk. Table 5 shows the STBLS risk level determination according to the three scoring parameters.

### 3.3. Classifier Configuration

For selecting the best classifier that is also relevant to the proposed input and output dataset, we considered the traditional ML methods that were already used for classifying skeletal data in previous research such as, Support Vector Machine (SVM) [39], Random Forest (RF) [40], K-Nearest Neighbors (kNN) [39], Linear Regressor [41], Logistic Regressor [40], and Artificial Neural Network (ANN) [25]. After implementing the mentioned classifiers for our data, we selected the results of RF and ANN classifiers to report in detail. Even though the remaining classifiers were implemented, acceptable accuracy rates could not be achieved due to insufficient training data, resulting in overfitting. This section introduces the classifiers used to identify individuals’ risk levels using the STBLS scoring system of the JOA. The features shown in Table 1 and Table 3 are used as the input values to the classifiers to predict the individual STBLS scores.

#### 3.3.1. Artificial Neural Network (ANN)

**Figure 5:**
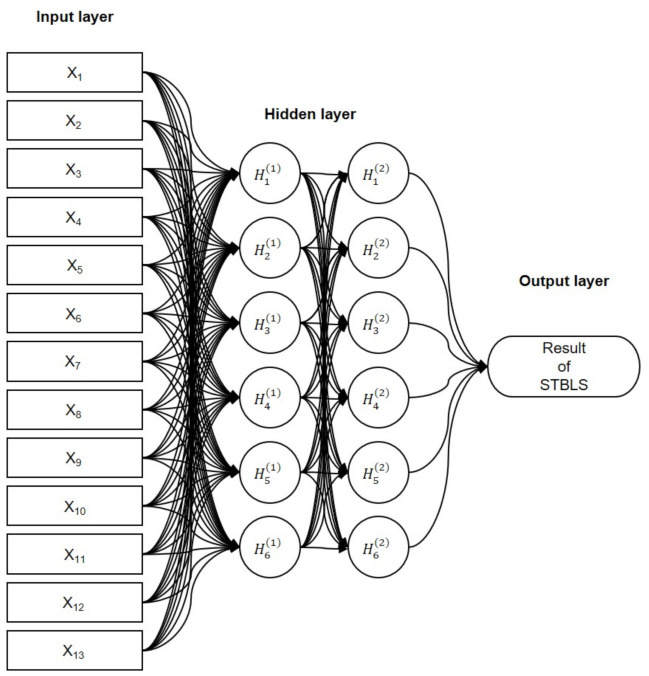
Combined Artificial Neural Network (ANN) for predicting STBLS score based on squat and one-leg standing measurement parameters.

Figure 5 shows the structure of the proposed neural network. Two hidden layers contain six nodes per layer, and the output layer uses one of the STBLS test scores. The activation function ($f(x_{i})$) used here is the Rectified-Linear-Unit function (ReLU).

(14) $$\begin{array}{c} f\left(x_{i}\right)=max\left(0,x_{i}\right)+0.01*min\left(0,x_{i}\right) \end{array}$$

(15) $$\begin{array}{c} F\left(x_{i}\right)=max\left(0,x_{i}\right)/x_{i}+0.01*min\left(0,x_{i}\right)/x_{i} \end{array}$$

$F(x_{i})$ represents the derivative of the activation function, $f(x_{i})$. The first step is forward propagation. Then, the first hidden layer, $H_{i}^{\left(1\right)}$ is calculated by using the input features $X_{i}$ (input layer containing 13 nodes) and weights $w_{ji}^{\left(0\right)}$. The second hidden layer, $H_{k}^{\left(2\right)}$, is calculated by using the first hidden layer $H_{j}^{\left(1\right)}$ and weights $w_{kj}^{\left(1\right)}$. Finally, the output *Y* is calculated using the hidden layer $H_{k}$ and the weights $w_{1k}^{\left(2\right)}$.

The initial values of the weights, $w_{ji}^{\left(0\right)}$, $w_{kj}^{\left(1\right)}$, and $w_{1k}^{\left(2\right)}$, use the initial value of “He”. “He” is set randomly from a normal distribution with a mean of 0 and a standard deviation of $\sqrt{2}/n$ for n parameters. Next, backpropagation is performed. The error $e^{\left(2\right)}$ between the output Y and the measured score *O* of the STBLS is calculated. Next, the contributions of the current hidden-layer weights $w_{1k}^{\left(2\right)}$ and $w_{kj}^{\left(1\right)}$ to the output error are calculated.

When the output error is small, the output parameters are determined by repeatedly processing forward propagation and error backpropagation. Hence, the updated weight parameters are used to predict the test score of STBLS for new users that are unknown to the trained classifiers. The number of learning epochs was 100,000, and the learning rate lr used for tuning the parameters was 0.005.

#### 3.3.2. Random Forest Regressor

In addition to ANN, we also considered using the Random Forest (RF) regressor to recognize our skeletal data-based parameters. Since our input data were paired with corresponding STBLS output scores, the supervised nature of RF regressor made it an apposite choice. RF regressors include the ensemble of a large number of decision trees which operate individually. Each decision tree predicts an output class, and the output class that is predicted by most number of trees is the final prediction of the classifier. The selection of optimal number of decision trees is essential for minimizing computational cost and for obtaining high accuracy simultaneously. In this work, we obtained the accuracy by varying the number of trees from 5 through 100.

## 4. Accuracy Evaluation

We performed a user study to determine the accuracy of our proposed risk assessment method. The study was designed by following the ethical regulations postulated by the Declaration of Helsinki. Informed consent was acquired from all subjects. The conditions for training the classifiers included the following: using squat features only ($X_{1}$ to $X_{9}$), using one-leg standing features only ($X_{10}$ to $X_{13}$), and using all features ($X_{1}$ to $X_{13}$). We used leave-one-out cross-validation for evaluating the accuracy of prediction.

### Methodology

Ten subjects aged 20 to 35 years without any reported history of ailments participated. The experiment was divided into three stages:

- STBLS scores: The three STBLS scores were first recorded and used as the classifiers’ outputs;
- Squat measurement: The subjects were instructed to perform a 1 min squat. The procedure is shown in Figure 1b;
- One-leg standing measurement: Subjects were instructed to maintain the one-leg standing posture for 70 s and maintain the lifted knee and hip joints, precisely at 90 degrees. The subjects were also asked to gaze at a magnet placed at eye level to ensure concentration. The procedure is shown in Figure 2b.

The squat and one-leg standing measurements were carried out within a six month period. Since a time gap was present between the two measurements for some subjects, we verified that there were no changes in their physical condition by recording STBLS scores during both measurements. The JOA STBLS assessment results, squat, and one-leg standing input features are shown in Table 6 and Table 7. The average scores of stand-up, 2-stride, and GLFS-25 were 6.93 ± 0.995, 1.44 ± 0.013, and 2.71 ± 5.91, respectively.

## 5. Results

**Figure 6:**
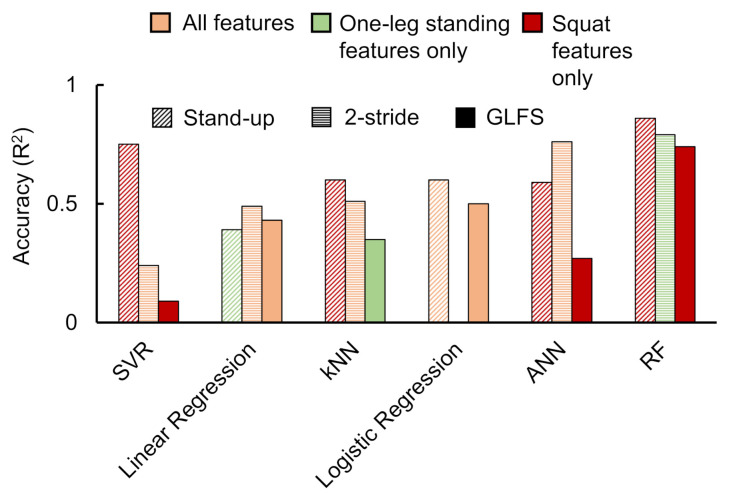
Comparing the accuracy of all classification methods. The accuracy values mentioned here indicate the highest value out of the three considered conditions of parameter selection as indicated by the type of color (all features, squat features only, and one-leg standing features only). The shades indicate the STBLS test scores considered (stand-up, 2-stride, and GLFS-25).

Figure 6 illustrates the best accuracies achieved for all considered classification approaches when implemented with the available data. The graph depicts the best accuracies for each classification parameter selection condition. From these data, it can be observed that the best performance was achieved in the case of ANN and RF classifiers. Owing to this observation, we selected these two classifier data to be reported in detail.

**Figure 7:**
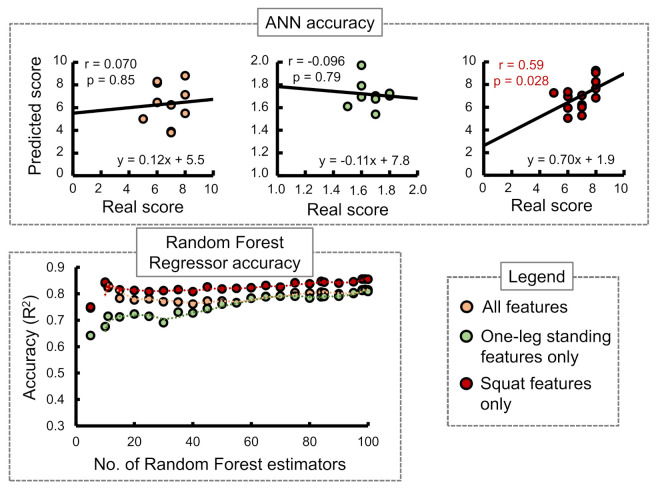
Comparing the accuracy of the ANN and Random Forest regressor in predicting the STBLS test stand-up score.

**Figure 8:**
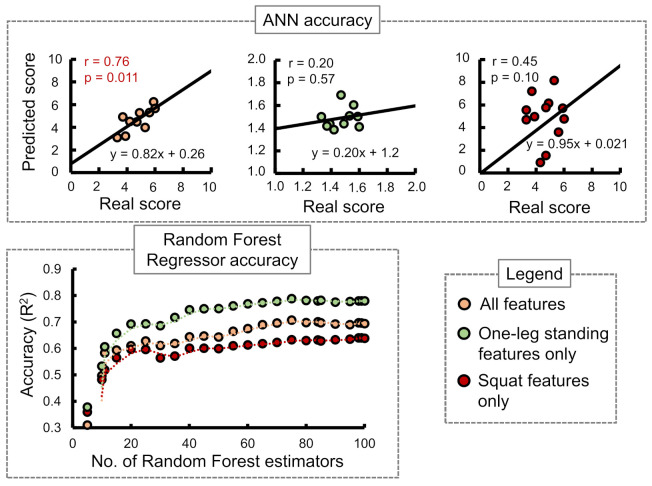
Comparing the accuracy of the ANN and Random Forest regressor in predicting the STBLS test 2-stride score.

**Figure 9:**
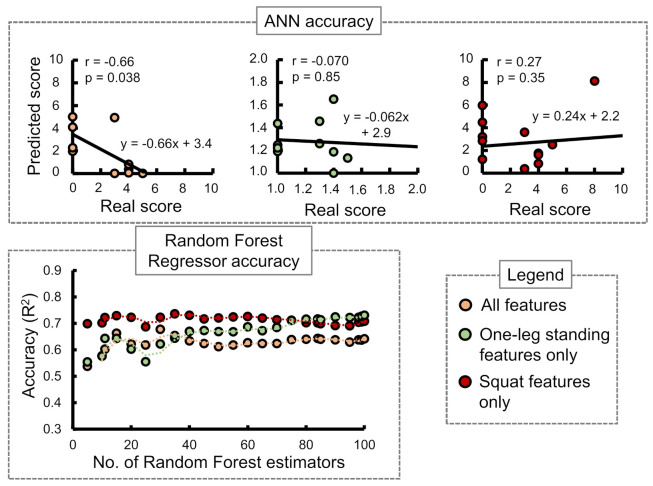
Comparing the accuracy of the ANN and Random Forest regressor in predicting the STBLS test GLFS-25 score.

- Stand-up score: Figure 7 shows the ANN and RF accuracies for the stand-up score. The ANN correlation coefficients while considering all input features, one-leg standing features only, and squat features only were observed as 0.07, −0.096, and 0.59, respectively. The best RF regressor accuracy ($R^{2}$) was obtained while considering squat features only followed by all features and one-leg standing features only.
- Two-stride score: Figure 8 shows the ANN and RF accuracies for the 2-stride score. The ANN correlation coefficients while considering all input features, one-leg standing features only, and squat features only were observed as 0.76, 0.20, and 0.45, respectively. The best RF regressor accuracy ($R^{2}$) was obtained while considering one-leg standing features only followed by all features and squat features only.
- GLFS-25 score: Figure 9 shows the ANN and RF accuracies for GLFS-25 score. The ANN correlation coefficients while considering all input features, one-leg standing features only, and squat features only were observed as −0.66, −0.070, and 0.27, respectively. The best RF regressor accuracy ($R^{2}$) was obtained while considering squat features only followed by one-leg standing features only and all features.

In case of the ANN accuracy, we observed that the maximum correlation coefficient between predicted and real scores while using only squat input parameters from $i_{1}$ to $i_{9}$ was 0.59. On the other hand, while using only one-leg standing parameter inputs from $i_{10}$ to $i_{13}$, no correlation between the predicted and actual scores was observed. However, while combining both training set inputs from $i_{1}$ to $i_{13}$, an apparent increase in the correlation coefficients was observed for stand-up and 2-stride scores specifically.

The accuracies of the RF regressor, on the other hand, indicated squat features to be the most suitable choice for predicting stand-up and GLFS-25 scores. One-leg standing features produced the highest accuracy in predicting the 2-stride score. Table 8 shows the best accuracies obtained for the three STBLS scores considering different input feature conditions.

The SVR method produced the high accuracy ($R^{2}$) during two instances, namely stand-up score prediction using all features (0.62) and squat features only (0.75). The SVR accuracies for the remaining conditions were not in an acceptable range. Moreover, even though we applied linear regression on our data and achieved accuracies in the range of 0.4 to 0.5, there were issues of over-fitting. kNN also showed signs of overfitting when low values of k were selected. Logistic regression was applied to classify stand-up and GLFS test scores only since it does not support non-integer values as the output layer. Selecting all features produced higher accuracy for this method.

## 6. Discussion and Future Direction

While using the ANN, we observed that the best correlation coefficient between predicted and real scores was 0.59 (moderate correlation) for identifying stand-up scores using the squat features only ($i_{1}$ to $i_{9}$). The one-leg standing features ($i_{10}$ to $i_{13}$) only were not useful for any score prediction. However, while combining both training set inputs ($i_{1}$ to $i_{13}$), an increase in the correlation coefficient to 0.76 (high correlation) was observed for the 2-stride score. Hence, it is crucial to train the algorithm by combining one-leg standing features and squat features to achieve improved accuracy. From the results obtained in this study, the stand-up and 2-stride test scores could be predicted with acceptable accuracy by choosing the appropriate input features. The quality of the observed correlation coefficients has been termed moderate or high according to previously published work on ANN prediction [42]. On the other hand, while using the RF regressor to perform the score predictions, we could achieve the highest accuracies ($R^{2}$) of 0.856, 0.788, and 0.736 for stand-up, 2-stride, and GLFS-25 test scores, respectively. Individual features of squat and one-leg standing were more efficient compared to the combined features. This behaviour can be associated with the observation made by previous literature that even small feature subsets are sufficient for achieving full base accuracy in the case of RF classifiers [43]. The STBLS test facilitates accurate identification of LS. The same has also been implemented through the proposed classifiers in this paper. This risk prediction method can help determine difficulty levels of therapeutic exercises deployed to pathological patients. On the other hand, this system can also evaluate healthy adults who may be unknowingly progressing towards LS.

Limitations were found in our current version of the estimation system. We could not achieve significantly high accuracy with the given conditions due to various possible reasons. The squat measurement sessions were held without limiting the squat depth, which may have caused variation in the data. In case of ANN, the real and predicted scores for GLFS-25 and stand-up tests had low correlation compared to 2-stride due to the use of integer values in the STBLS scoring. Moreover, the GLFS-25 questionnaire mainly depends on the participants’ subjective responses; hence, the prediction of this score with motion measurement was challenging. The target users of our work are not limited to the elderly, which is why lower limb skill assessments were performed for young subjects in the current paper. The inclusion of elderly and patient data was a necessary step to validate the proposed method of parameter selection and classification. Therefore, conclusions cannot be drawn about how the classifier will perform on non-healthy subject data. Future investigations also includes reconsidering subjective or questionnaire-based risk level indicators.

We obtained acceptable classifier accuracy for young subjects. In order to further improve the efficiency and variation in training data, expanding the number and variation of subjects in terms of age, gender, and physical fitness is our next step. A large dataset will open up the possibility of testing additional classifiers and retesting the classifiers that are already tested and mentioned in this paper. Commonly used deep ML methods on skeletal data such as Convolutional Neural Network (CNN) [44] and Recurrent Neural Network (RNN) [45] will also be attempted in order to verify their usability with the proposed and additional set of skeletal parameters for skill assessment. Skeletal features that were not considered in this paper will also be tested for usability in these classifiers.

An important prospect of this research is its implementation as a personal health monitoring or assessment module which can be used without therapist assistance. The usage of Intel RealSense comes with the added advantage of being convenient to install and inexpensive compared to similar depth cameras. After the acquisition of additional training data, integration of the tracking system and an adequately trained classifier will provide us with an independently usable skill assessment tool. However, to ensure medical safety and to make the system usable by patients and elderly, we plan to integrate algorithms to detect unsafe postures and to stop the session in order to avoid injury. In addition, a graphical user interface (GUI)-based application will be designed and developed to allow users to interact with the assessment tool and store their data for future reference. This tool will be especially helpful for individuals who are unaware that they are approaching towards the onset of Locomotive Syndrome. Monitoring their lower limb constantly will motivate them to stay physically active. This type of skill assessment can also be integrated with rehabilitation robots with control strategies, which can manipulate the amount of assistive force according to the identified risk level [46]. Rehabilitation robots and exoskeleton suits for training the elderly and other pathological individuals are gradually being accepted, and weighted models for assessing individuals’ ability and training them based on their skill levels would optimize the process of rehabilitation. Therefore, the proposed risk assessment method can also be utilized to manipulate the assistive or resistive forces delivered to healthy and pathological users while taking part in workout sessions and therapeutic exercises.

## 7. Conclusions

As the current times challenge us with sedentary lifestyles and reduced mobility, it is more likely that adults will progress towards immobility disorders such as the *Locomotive Syndrome (LS)*. The only way to combat this is its early detection and taking necessary measures. Conventional methods of identifying LS in adults are quite useful but are subjective and require several resources such as adjustable height stool, weighing scale, and measuring tape. Therefore, to make this process easier, we introduced an ML-based system that estimates the LS risk level. This estimation tool uses 13 parameters acquired from squat and one-leg standing exercises as input layer data. These parameters were obtained by processing raw skeletal data recorded through the Intel RealSense depth camera. We predicted the stand-up and 2-stride scores of the STBLS test with correlation coefficients of 0.59 and 0.76 between the real and predicted data, respectively, when using an ANN. In addition, an RF regressor could predict the stand-up, 2-stride, and GLFS-25 scores with accuracies of 0.856, 0.788, and 0.736, respectively.

## Figures and Tables

**Table 1:** Parameters considered for the squat motion measurement. Here, the units of angle, shakiness, and time-based parameters are degree, meter, and second, respectively.

Index	Parameters
$\theta_{lkf}$	Left knee flexion angle
$\theta_{rkf}$	Right knee flexion angle
$\theta_{lke}$	Left knee extension angle
$\theta_{rke}$	Left knee extension angle
$s_{lk}$	Left knee shakiness
$s_{rk}$	Right knee shakiness
$\theta_{lhf}$	Left hip flexion angle
$\theta_{rhf}$	Right hip flexion angle
$\theta_{lhe}$	Left hip extension angle
$\theta_{rhe}$	Right hip extension angle
$t_{e}$	Extension time
$n_{s}$	Number of squats

**Table 2:** Inputs to the neural network considered for the squat motion measurement.

Index	Features
$X_{1}$	Left knee angular displacement
$X_{2}$	Right knee angular displacement
$X_{3}$	Left hip angular displacement
$X_{4}$	Right hip angular displacement
$X_{5}$	Left knee lateral displacement
$X_{6}$	Right knee lateral displacement
$X_{7}$	COM smoothness
$X_{8}$	Squat extension rate
$X_{9}$	Squat completion rate

**Table 3:** Parameters considered for the one-leg standing measurement. The units for time and position-based parameters are second and meter, respectively. Abbreviations used in this table: Dominant Leg (DL); Non-Dominant Leg (NDL).

Index	Parameters
$t_{dl}$	Standing time (DL)
$t_{ndl}$	Standing time (NDL)
$x_{dl}$	X axis position of waist (DL)
$y_{dl}$	Y axis position of waist (DL)
$z_{dl}$	Z axis position of waist (DL)
$x_{ndl}$	X axis position of waist (NDL)
$y_{ndl}$	Y axis position of waist (NDL)
$z_{ndl}$	Z axis position of waist (NDL)

**Table 4:** Inputs to the neural network considered for the one-leg standing measurement.

Index	Features
$X_{10}$	Standing time (DL)
$X_{11}$	Standing time (NDL)
$X_{12}$	Total trajectory length (DL)
$X_{13}$	Total trajectory length (NDL)

**Table 5:** Using the Short Test Battery Locomotive Syndrome (STBLS) test parameters to determine the Risk Level (RL) in individuals. The STBLS test includes stand-up, 2-stride, and Geriatric Locomotive Function Scale (GLFS-25) scores [38].

RL	Stand-Up Test	2-Stride Test	GLFS-25
0	$O_{1}>4$	$O_{2}\geq 1.3$	$O_{3}<7$
1	$2<O_{1}\leq 4$	$1.1\leq O_{2}<1.3$	$16>O_{3}\geq 7$
2	$O_{1}\leq 2$	$O_{2}<1.1$	$O_{3}\geq 16$

**Table 6:** Results of the input features from squat measurement ($X_{1}$ to $X_{9}$) for 10 subjects.

Subject	$X_{1}$	$X_{2}$	$X_{3}$	$X_{4}$	$X_{5}$	$X_{6}$	$X_{7}$	$X_{8}$	$X_{9}$
A	0.385	0.335	0.199	0.261	1.000	1.000	0.850	0.826	0.833
B	0.869	0.987	0.942	0.942	1.000	1.000	0.570	0.152	0.667
C	0.752	0.823	0.503	0.306	0.648	0.445	0.697	0.247	0.917
D	0.742	0.581	0.508	0.650	1.000	1.000	0.569	0.807	0.917
E	0.244	0.336	0.113	0.203	0.838	0.820	0.747	0.334	0.917
F	0.395	0.684	0.773	0.601	1.000	1.000	0.294	0.690	0.833
G	0.929	0.730	0.861	0.987	1.000	1.000	0.508	0.476	0.917
H	0.841	0.847	0.624	0.627	1.000	1.000	0.986	0.098	0.667
I	0.406	0.620	0.707	0.423	0.745	0.565	0.345	0.882	0.750
J	0.778	0.758	0.671	0.508	0.888	0.876	0.824	0.118	1.000

**Table 7:** Results of the STBLS assessment and one-leg standing measurement ($X_{10}$ to $X_{13}$) for 10 subjects.

Subject	Stand-Up	2-Stride	GLFS-25	$X_{10}$	$X_{11}$	$X_{12}$	$X_{13}$
A	6	1.59	5	1	1	0.285	0.307
B	5	1.49	3	1	0.874	0.291	0.291
C	7	1.33	4	1	1	0.741	0.341
D	7	1.53	4	1	1	0.794	0.378
E	6	1.47	3	1	1	0.849	0.729
F	8	1.60	0	1	1	0.722	0.437
G	8	1.43	3	1	1	0.528	0.202
H	6	1.39	0	1	1	0.634	0.228
I	8	1.42	0	1	1	0.399	0.377
J	8	1.47	0	1	1	0.205	0.414

**Table 8:** Maximum accuracies ($R^{2}$) achieved through the Random Forest regressor.

STBLS Score	Squat Features	One-Leg Standing Features	All Features
Stand-up	0.856	0.813	0.84
2-stride	0.64	0.788	0.707
GLFS-25	0.736	0.731	0.678

## Data Availability

The data used in this study will be made available upon requesting the corresponding author with appropriate reasoning.

## Abbreviations

The following abbreviations are used in this manuscript:

ANN	Artificial Neural Network
BBS	Berg Balance Scale
CNN	Convolutional Neural Network
COM	Center Of Mass
DL	Dominant Leg
FFANN	Feed forward ANN
FPS	Frames Per Second
GANN	Genetic Algorithm Neural Network
GLFS	Geriatric Locomotive Function Scale
IMU	Inertial Measurement Unit
JOA	Japanese Orthopedic Association
kNN	k-Nearest Neighbors
LS	Locomotive Syndrome
ML	Machine Learning
NDL	Non Dominant Leg
PCA	Principal Component Analysis
ReLU	Rectified Linear Unit
RL	Risk Level
RNN	Recurrent Neural Network
SIMS	Smart Insole Measurement System
SOM	Self Organized Maps
SPPB	Soft Physical Performance Battery
STBLS	Short Test Battery Locomotive Syndrome
SVM	Support Vector Machine
SVR	Support Vector Regressor
Tinetti-POMA	Tinetti Performance Oriented Mobility Assessment
TUG	Timed Up and Go
VGRF	Vertical Ground Reaction Force
